# The Role of Gut Microbiome in Mild Cognitive Impairment: A Twin Study

**DOI:** 10.3390/medicina62061106

**Published:** 2026-06-06

**Authors:** Aliz Persely, Marton Piroska, Luca Zoldi, Beatrix Beszedics, Janos Juhasz, Nora Makra, Zsuzsanna A. Dunai, Dora Szabo, David Laszlo Tarnoki, Adam Domonkos Tarnoki

**Affiliations:** 1 Medical Imaging Centre, Semmelweis University, 1082 Budapest, Hungary; piroskamarton94@gmail.com (M.P.); luca.zoeldi@gmail.com (L.Z.); beszedics.beatrix@gmail.com (B.B.); tarnoki4@gmail.com (D.L.T.); tarnoki2@gmail.com (A.D.T.); 2 Institute of Medical Microbiology, Semmelweis University, 1089 Budapest, Hungarynora.makra@indamail.hu (N.M.); zsuzsanna.dunai@gmail.com (Z.A.D.); szabo.dora@semmelweis.hu (D.S.); 3 Faculty of Information Technology and Bionics, Pazmany Peter Catholic University, 1083 Budapest, Hungary; 4 HUN-REN-SU Human Microbiota Research Group, 1052 Budapest, Hungary; 5 Department of Neurosurgery and Neurointervention, Semmelweis University, 1085 Budapest, Hungary

**Keywords:** gut microbiome, alpha diversity, dysbiosis, gastrointestinal tract, dementia, Alzheimer’s disease, cognitive dysfunction, neurodegeneration

## Abstract

*Background and Objectives*: Recent studies have revealed the potential roles of gut microbiota and microbial metabolites in influencing mild cognitive impairment (MCI) and Alzheimer’s disease via the gut–brain axis. This relationship has not yet been investigated in monozygotic twin pairs, which represent an ideal model for minimizing genetic confounding. *Materials and Methods*: Seven twin pairs discordant for ACE and 15 for MoCA were enrolled. Stool samples were subjected to 16S ribosomal RNA-based microbiome analysis. *Results*: No significant differences in alpha or beta diversity were observed between MCI-discordant twin pairs at the genus or family level. The most robust finding was a significantly lower abundance of *Lachnospiraceae* in MCI-affected twins, identified independently by ANCOM-BC and LEfSe. Additional exploratory findings included higher abundances of *Sutterella*, *Succinivibrio*, *Odoribacter*, and *Ruminococcus*. However, several taxa showed opposing patterns between ACE- and MoCA-derived cohorts, highlighting the methodological impact of cognitive instrument selection. *Conclusions*: The convergent reduction of *Lachnospiraceae* across two independent analytical methods represents the most substantive finding. The remaining results are exploratory, limited by small sample size, restricted statistical power, and lack of availability to fully control for dietary habits, physical activity, and medication use. Validation in larger longitudinal twin cohorts with a standardized cognitive assessment is warranted.

## 1. Introduction

Dementia is a major public health problem, with an estimated 50 million people worldwide currently living with the condition, a number projected to triple by 2050 [1]. Several types of dementia are recognized, which may develop independently or concurrently. Prevalence increases markedly with age, affecting approx. 20% of the population over 85 years of age [2]. Alzheimer’s disease (AD) is the leading cause of dementia, accounting for approximately two-thirds of all cases. Risk factors for cognitive decline include genetic predisposition, lifestyle factors, and other pathologies and comorbidities that become more prevalent with advancing age [3]. Among lifestyle factors, physical inactivity, poor nutrition, and smoking have been most consistently associated with elevated dementia risk. Vascular comorbidities, including hypertension, diabetes mellitus, and stroke, are also recognized contributors to the development and severity of various dementias [4].

Mild cognitive impairment (MCI) represents a clinical state between normal cognition and dementia, in which both progression and regression may occur, and which is not equivalent to clinical dementia. Originally considered as a precursor to AD, MCI is now conceptualized as a heterogeneous condition that may progress to multiple dementia subtypes. Accordingly, several classification systems have been proposed, including the amnestic and non-amnestic MCI subtypes [5]. Moreover, AD itself is similarly heterogeneous and is increasingly referred to as the Alzheimer’s Disease Spectrum; co-pathologies may further impact its clinical presentation [6]. Approximately 10–12% of MCI patients progress to AD yearly. However, progression is neither universal nor inevitable. A recent study reported that neuropsychological tests and brain magnetic resonance imaging-related markers could accurately predict the progression from MCI to AD using machine learning, especially in a short period of time [7,8].

Recent studies have revealed the potential role of the gut microbiota and microbial metabolites in influencing MCI and AD and AD’s progression. It has been shown that behavioral and cognitive phenotypes are affected by the gut microbiome, which is connected to the central nervous system through the gut–brain axis [9,10]. Microbiome dysbiosis has been associated with increased intestinal permeability and systemic inflammation, which may promote neurodegeneration [9]. A randomized controlled trial showed that cognitive performance improved in parallel with changes in gut bacterial profile [11]. Furthermore, gut dysbiosis has been detected in MCI and AD in the systematic review and meta-analysis of 34 case-control studies [12].

To date, knowledge regarding the relationship between MCI and the intestinal microbiota remains limited. The present study is the first to investigate this association in monozygotic twin pairs, a design that inherently minimizes the influence of genetic background and shared early-life environmental factors, allowing observed microbiome differences to be more confidently attributed to non-shared environmental influences or disease-associated biological processes, advantages difficult to achieve in conventional case-control or population-based studies. However, causality cannot be established from the present data, and the small sample size limits statistical power and generalizability.

## 2. Materials and Methods

### 2.1. Study Participants

A total of 113 Caucasian subjects (56 monozygotic twin pairs, including one triplet set) registered in the population-based Hungarian Twin Registry [13] were invited to participate in the study.

Exclusion criteria included acute infection within three weeks prior to enrollment, acute respiratory, cardiac, or renal failure, underlying malignancy, inflammatory bowel disease, a history of carotid surgery, diagnosed neurological disorders (e.g., stroke, multiple sclerosis), and depression. From the initial 56 MZ twin pairs, participants younger than 65 years were subsequently excluded. The triplet set was also excluded owing to a neurological diagnosis in one member.

The study was approved by the local Ethical Committee (Semmelweis University TUKEB 217/2021). All participants provided written informed consent, and the study was conducted in accordance with the principles of the Declaration of Helsinki. Zygosity was determined using a validated seven-item self-reported questionnaire [14]. All examinations were performed at the Semmelweis University Medical Imaging Centre, Department of Neuroradiology in Budapest, Hungary.

Comprehensive data on general health status, medical history, current medication use, dietary habits, and risk factors (including smoking, hypertension, hyperlipidemia, and diabetes mellitus) were collected via a structured questionnaire. Body weight and height were assessed, and body mass index was calculated. Based on self-reported medication records, none of the included participants reported current use of laxatives, antibiotics, probiotics, or anti-inflammatory medications. Cognitive and psychiatric status were assessed through personal interview-based questionnaires. Neurocognitive function was evaluated using the Addenbrooke’s Cognitive Examination (ACE) and the Montreal Cognitive Assessment (MoCA). The remaining participants were categorized according to cognitive status: concordant pairs, in which both twins met criteria for cognitive decline, and discordant pairs, in which only one twin was affected.

### 2.2. Questionnaires

The ACE test (ACE-III) is a cognitive screening test. was administered to the participants. This test was originally developed as an improved extension of the Mini-Mental State Examination [15] and is used as a screening and follow-up instrument for patients, for example, with frontotemporal dementia and primary progressive aphasia [16,17].

The ACE test investigates five cognitive domains: attention/orientation, memory, language, verbal fluency, and visuospatial skills. At the recommended cut-off scores of 88 and 83, the ACE was reported to have good sensitivity and specificity for identifying different forms of dementia and other impairments of memory and judgement (0.93 and 0.71; 0.82 and 0.96, respectively) [15,18].

In our study, we used the Hungarian validated version of the ACE test [19]. Regarding the Hungarian version, the following categories are recommended:

- 85–100 points: No cognitive disorder;
- 84–75 points: Mild cognitive impairment;
- 74–60 points: Moderate cognitive impairment;
- below 60 points: Severe cognitive impairment.

The Montreal Cognitive Assessment (MoCA) was also used, which is a widely used screening tool for detecting MCI and dementia [20]. The symptom groups assessed by the MoCA test were various elements of memory, spatial, executive functions, language skills, attention, abstraction, delayed recall, and orientation. By evaluating the test, the following classifications can be determined:

- 26–30 points: No cognitive disorder;
- 18–25 points: Mild cognitive impairment;
- 10–17 points: Moderate cognitive impairment;
- 0–9 points: Severe cognitive impairment.

Therefore, the discordance for cognitive impairment was set for 84 points concerning ACE and 25 for MoCA scores.

Unlike the MoCA, which incorporates a standardized, built-in education-based score correction, adding one point for participants with 12 or fewer years of formal education, the ACE does not apply an equivalent automatic adjustment, although education-stratified cut-off values have been proposed in some validation studies.

### 2.3. Stool Sample Collection, Processing

Stool sample collection, processing, and subsequent bioinformatics and statistical analyses were conducted according to standardized protocols, following the manufacturers’ instructions for all reagents and collection devices used, as detailed below. Participants received a comprehensive instruction manual accompanied by a flow chart outlining the importance of rapid sample return, avoidance of external contamination, adequate sample quantity, and correct storage and packaging [21].

Stool samples were collected using DNA/RNA Shield Fecal Collection Tubes (Zymo Research, Irvine, CA, USA), which contain a stabilizing shield solution that preserves microbial nucleic acids at room temperature for extended periods. To minimize contact between the stool specimen and the toilet bowl prior to collection, participants were provided with Fe-Col Faecal Collection Paper (Alpha Laboratories, Eastleigh, UK), placed on the toilet seat during defecation. The DNA/RNA Shield Fecal Collection Tubes and Fe-Col Faecal Collection Paper were used according to the respective manufacturers’ protocols. Following collection, samples were stored at −80 °C until further processing. Although the precise interval between sample collection and freezing could not be reconstructed for all participants, this period did not exceed one week in any case. Notably, the stabilizing shield solution present in the collection tubes is designed to maintain sample integrity at room temperature for up to one year, mitigating potential degradation during transport.

Genomic DNA was extracted using the ZymoBIOMICS DNA Miniprep Kit (Zymo Research, Irvine, CA, USA) according to the manufacturer’s protocol. Library preparation targeting the V3–V4 hypervariable region of the 16S ribosomal RNA gene was performed following the Illumina 16S Metagenomic Sequencing Library Preparation protocol [21]. Prior to sequencing on an Illumina MiSeq platform, libraries were indexed with unique dual indices, quality-assessed using an Agilent 2100 Bioanalyzer (Agilent Technologies, Santa Clara, CA, USA), and subsequently pooled in equimolar ratios.

### 2.4. Bioinformatics and Statistical Analysis

Read preprocessing was performed using FastQC v0.12.1 for quality assessment. Trimmomatic v0.39 was used for quality trimming, repair.sh for read re-pairing, and BBmerge.sh from the BBTools v39.38 suite for read merging, with the following parameters: error rate 0.1, indels true, adapter overlap 3, quality trimming true, trim from 5′ end 0, trim 3′ end to read length 0, sliding window size 4, required quality for sliding window 12, leading quality 3, trailing quality 3, minimum length 30, average quality across read 0, merge read pairs true, strictness default, re-pair reads true.

Taxonomic classification was performed using QIIME2 (2022 release) [22] with the following parameters: clustering algorithm closed reference, OTU reference database SILVA v138 [23], chimera removal false, phylogenetic tree MAFFT, taxonomy classification method sklearn, sklearn database SILVA v138 full length, percent identity 0.97, minimum Phred quality score 20, minimum abundance for bar plots 200.

Alpha diversity was estimated using the Shannon entropy index at the genus and family levels; group comparisons between MCI-affected and unaffected individuals were performed within the QIIME2 analytical framework as implemented on the Nephele cloud platform [24]. Beta diversity was assessed using Bray–Curtis dissimilarity, which accounts for the presence, absence, and relative abundance of taxa, and weighted UniFrac dissimilarity, which additionally incorporates the phylogenetic relationships between taxa. Both metrics were visualized by two-dimensional principal coordinate analysis (PCoA). Taxa with a relative abundance below 1% were excluded from all analyses. PERMANOVA was performed using Bray–Curtis dissimilarity at genus and family levels with 999 permutations to test for differences in overall microbial community structure between MCI-affected and unaffected individuals within discordant twin pairs. Differential abundance was assessed using ANCOM-BC v3.16 and LEfSe v1.1.2. ANCOM-BC v.316 results are reported as log-fold changes and adjusted *p*-values (q-values; significance threshold q < 0.05; suggestive q < 0.1). LEfSe was applied with a significance threshold of *p* < 0.1 and a minimum LDA score of 1.0 (log_10_).

The benefits of twin design were utilized during the statistical analysis by comparing the members of twin pairs with each other instead of comparing the unrelated members of the different groups. This method helps decrease the unwanted component of variability between the groups caused by, for example, the different genetic backgrounds of the members.

## 3. Results

Of the total cohort, seven twin pairs discordant for ACE score and 15 twin pairs discordant for MoCA score, in whom microbiome data were available for both members, were included in the analysis (Table 1). Table 1 presents the baseline demographic and clinical characteristics of the selected twin pairs. No statistically significant differences were observed between the two groups. The two groups partially overlapped but were not identical.

**Figure 1:**
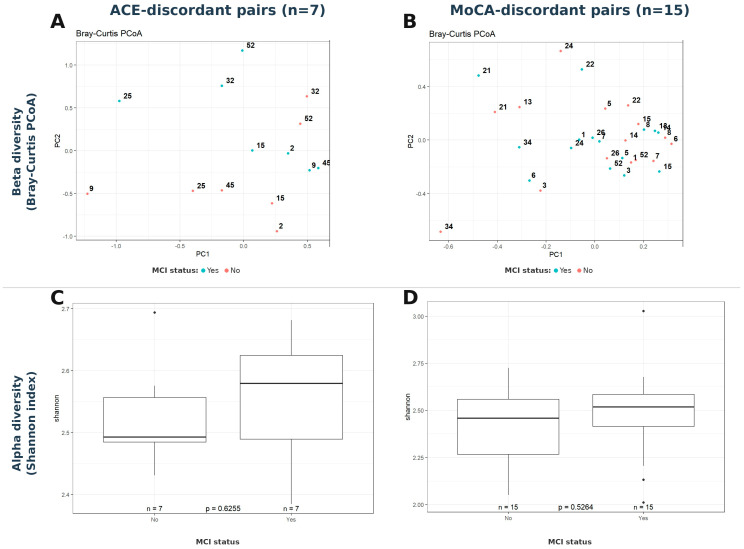
Gut microbiota diversity in MCI-discordant monozygotic twin pairs at genus level. Panels (**A**,**B**) display beta diversity assessed by Bray–Curtis dissimilarity-based principal coordinates analysis (PCoA) in ACE-discordant (*n* = 7) and MoCA-discordant (*n* = 15) twin pairs, respectively. Each point represents one individual sample; numbers indicate twin pair identifiers. Blue points represent MCI-affected twins; red points represent unaffected twins. Panels (**C**,**D**) display alpha diversity measured by the Shannon index for the corresponding groups. The horizontal line within each box represents the median. Box boundaries indicate the interquartile range (IQR); whiskers extend to 1.5× IQR; dots indicate outliers. No significant differences in alpha or beta diversity were observed between affected and unaffected twins in either cohort (all *p* > 0.05). PERMANOVA results are presented in Figure 2.

No significant differences in alpha diversity (Shannon index) were observed between MCI-discordant twin pairs at the genus level (ACE: *p* = 0.6255; MoCA: *p* = 0.5264) or family level (ACE: *p* = 0.605; MoCA: *p* = 0.423). Beta diversity was assessed using Bray–Curtis dissimilarity and weighted UniFrac dissimilarity and visualized by a principal coordinates analysis (PCoA). No distinct clustering by MCI status was observed at either the genus or family level using either dissimilarity metric (Figure 1; Supplementary Figure S1).

**Figure 2:**
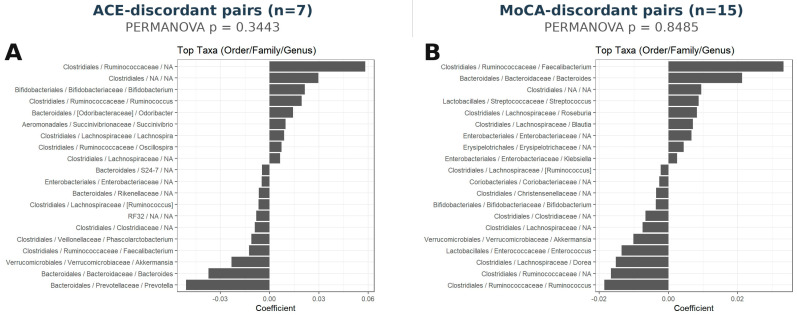
Taxa contributing most to gut microbial community dissimilarity between MCI-discordant MZ twin pairs, identified by PERMANOVA at genus level. Panel (**A**): ACE-discordant pairs (PERMANOVA *p* = 0.3443). Panel (**B**): MoCA-discordant pairs (PERMANOVA *p* = 0.8485). Bars represent PERMANOVA coefficients. Positive values indicate higher abundance in MCI-affected twins, and negative values indicate higher abundance in unaffected twins. Results did not reach statistical significance in either cohort.

A PERMANOVA based on Bray–Curtis dissimilarity at the genus level revealed no significant difference in microbial community structure between MCI-affected and unaffected twins in either cohort (ACE: *p* = 0.3443; MoCA: *p* = 0.8485); the results at the family level were similarly non-significant. Although overall community composition did not differ significantly at any taxonomic level or by either metric, *Clostridiales* and *Ruminococcaceae* contributed most to the observed variation in both cohorts at the genus level, while *Bacteroides* was among the leading contributors in the MoCA-discordant group (Figure 2).

**Figure 3:**
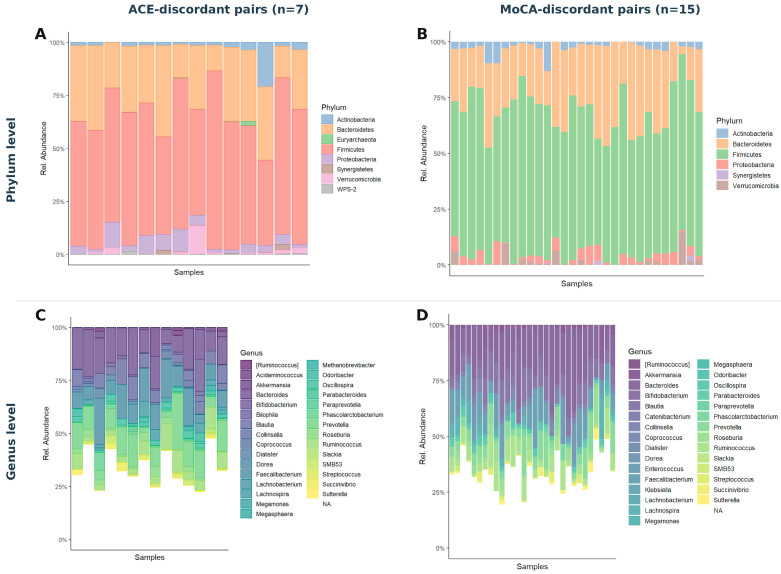
Relative abundance of gut microbial taxa in MCI-discordant monozygotic twin pairs at phylum and genus level. Stacked bar charts illustrate the relative abundance of gut microbial taxa in cognitively discordant MZ twin pairs, stratified by cognitive assessment tool and taxonomic level. Panels (**A**,**B**) display phylum-level composition in twin pairs discordant for the Addenbrooke’s Cognitive Examination (ACE; *n* = 7 pairs) and the Montreal Cognitive Assessment (MoCA; *n* = 15 pairs), respectively. Panels (**C**,**D**) display the corresponding genus-level composition for the same groups. Each bar represents one individual sample. Cognitively affected and unaffected twins are not distinguished within the bars; pairwise comparisons were performed at the statistical analysis level. Taxa with a relative abundance below 1% were excluded from the analysis. Colors represent distinct microbial taxa as indicated in the respective legends.

Taxa with a relative abundance below 1% were excluded from the analysis. The cognitively affected twin was compared with their unaffected twin. The relative abundance of microbial taxa across taxonomic levels, stratified by ACE and MoCA scores, is presented in Figure 3.

An Analysis of Composition of Microbiomes with Bias Correction (ANCOM-BC) was applied to identify taxa with significantly different relative abundances between MCI-affected and unaffected twins. Based on adjusted *p*-values (q-values), two taxa showed notable differences in the ACE-discordant group (Table 2). *Lachnospiraceae* demonstrated significantly lower abundance in MCI-affected twins (log-fold change = −0.041; *p* = 0.00038; q = 0.049). *Alphaproteobacteria* showed a trend toward lower abundance meeting the predefined threshold for suggestive significance (log-fold change = −0.009; *p* = 0.00148; q = 0.095; q < 0.1). No taxa reached significance thresholds in the MoCA-discordant group.

**Figure 4:**
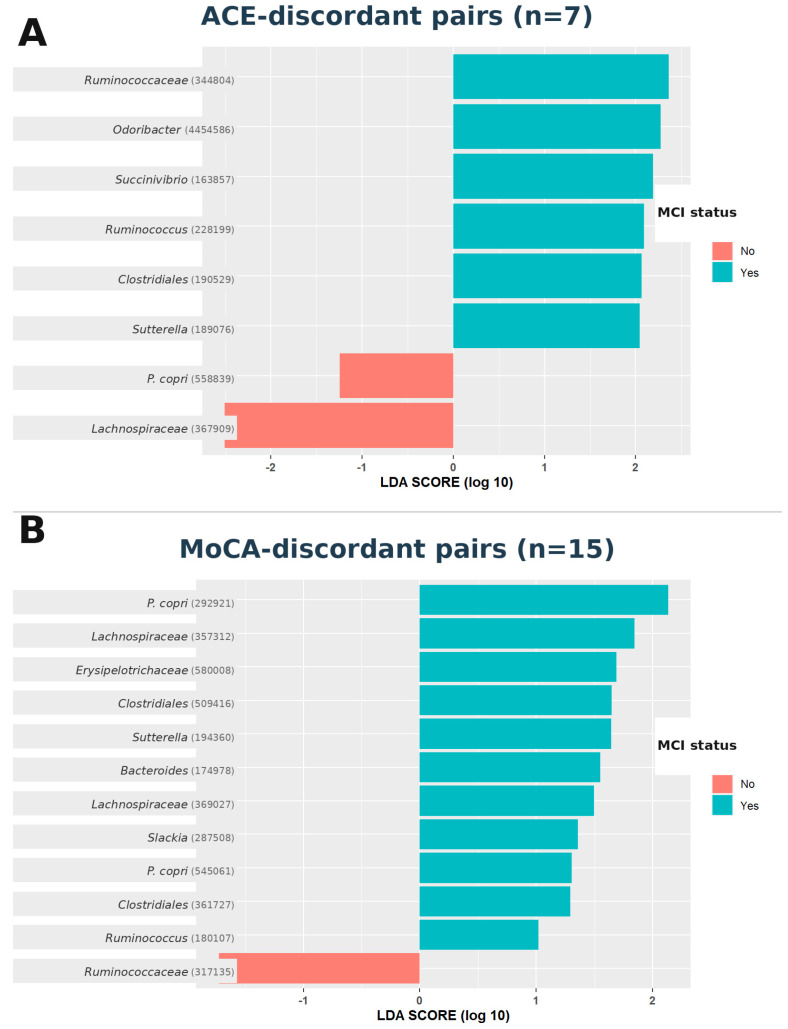
Differentially abundant gut microbial taxa identified by LEfSe analysis in MCI-discordant monozygotic twin pairs. Panel (**A**) shows results for ACE-discordant pairs (*n* = 7). Panel (**B**) shows results for MoCA-discordant pairs (*n* = 15). Horizontal bars represent the LDA score (log_10_), reflecting the effect size of each taxon. Blue bars indicate higher abundance in MCI-affected twins; red bars indicate lower abundance in MCI-affected twins (i.e., higher abundance in unaffected twins). The significance threshold for non-parametric tests was *p* < 0.1, with a minimum LDA score of 1.0 (log_10_). Taxon names are shown in italics; parenthetical numbers indicate taxon identifiers in the reference database. Taxa are ordered by LDA score magnitude within each panel. The numbers displayed in the figure correspond to taxon reference database identifiers, not quantities. They serve as labels to identify individual taxa in the reference database and do not represent numerical values or counts.

LEfSe analysis was performed to identify differentially abundant taxa between MCI-affected and unaffected twins, applying a significance threshold of *p* < 0.1 and a minimum LDA score of 1.0 (log_10_). The results are presented in Table 3 and Figure 4.

In ACE-discordant pairs, a lower abundance of *Lachnospiraceae* and *Prevotella copri* was detected in MCI-affected twins, alongside a higher abundance of *Sutterella*, *Ruminococcus*, *Succinivibrio*, *Odoribacter*, *Clostridiales*, and *Ruminococcaceae*. In MoCA-discordant pairs, a lower abundance of *Ruminococcaceae* was observed, while a higher abundance of *Ruminococcus*, *Clostridiales*, *Prevotella copri*, *Slackia*, *Lachnospiraceae*, *Bacteroides*, *Sutterella*, and *Erysipelotrichaceae* was detected in MCI-affected twins. An overlap between the two cohorts was observed for *Sutterella*, *Ruminococcus*, and *Clostridiales*, with all showing higher abundance in MCI-affected twins, albeit represented by different taxonomic entities. *Ruminococcaceae* was identified in both cohorts, but with opposing directions: higher abundance in ACE-discordant and lower abundance in MoCA-discordant pairs.

## 4. Discussion

To our knowledge, this is the first twin study to investigate the association between gut microbiota composition and mild cognitive impairment using both the Addenbrooke’s Cognitive Examination and the Montreal Cognitive Assessment. While discordant monozygotic twin studies are increasingly employed in microbiome research, their application to neurological conditions remains limited, and no prior study has examined gut microbiome changes in the context of cognitive decline using this design.

The MZ twin design offers a methodological advantage that is difficult to replicate in conventional study designs. MZ twins share approximately 100% of their genome and are exposed to highly similar early-life environmental conditions. Over time, however, phenotypic discordance emerges, driven in part by epigenetic changes and post-natal environmental differences. By inherently minimizing the influence of genetic background and shared early-life environmental factors, this design may allow observed microbiome differences between twins to be more confidently attributed to non-shared environmental influences, epigenetic modifications, or disease-associated biological processes. Such an approach may support the identification of individual-level factors relevant to patient care [25].

Over the past decade, the gut–brain axis has attracted considerable attention in neurological research, yet its precise role in the pathogenesis of neurological disorders, including dementia, remains incompletely understood. The gut microbiota can influence the gut–brain axis through multiple direct and indirect pathways, including neural, immune, endocrine, and metabolic mechanisms. This complex system is further shaped by numerous factors across the lifespan, including mode of delivery, breastfeeding, prematurity, environmental exposures, host genetics, antibiotic use, and maternal infection, stress, or obesity, as well as diet. The Irish ELDERMET study [26] suggested that greater microbiota diversity may be associated with better health outcomes, though this relationship requires further investigation.

Cognitive impairment and dementia, particularly AD, represent a growing global health burden. AD, the most common cause of dementia, is a neurodegenerative disorder characterized by progressive accumulation of amyloid-β plaques and tau deposits, typically presenting after the age of 65 with memory impairment. Although neuropathological changes precede clinical symptoms by years, timely diagnosis remains challenging. MCI has traditionally been regarded as a precursor to AD, but this concept has evolved, as MCI may progress to other dementia subtypes and may arise from heterogeneous etiologies, adding further complexity to the understanding of dementia pathophysiology. The gut–brain axis has been proposed as a potential contributor to neurodegeneration through neuroinflammation and amyloid deposition. However, demonstrating this relationship in humans remains difficult. While several studies have reported associations between gut microbiome alterations and dementia, findings have been mostly inconsistent, and few unambiguous conclusions can be drawn from the current literature [26,27].

In the present study, MCI-discordant MZ twin pairs were analyzed to minimize the confounding influence of genetic factors and focus on environmental and biological determinants of gut microbiota composition. Cognitive discordance was defined using two validated neuropsychological tests: ACE and MoCA, and the two resulting subgroups were analyzed separately. No significant differences in potential risk factors were identified between the two groups.

Neither alpha nor beta diversity differed significantly between MCI-affected and unaffected twins in either cohort, at either the genus or family level, or using either Bray–Curtis or weighted UniFrac dissimilarity. Reduced microbiota diversity has been reported in AD [27,28,29]. Also, several groups reported significant beta-diversity alterations in MCI and AD cohorts [11,30,31,32,33,34], while Nagpal et al. [35] and Ueda et al. [36] similarly failed to detect significant beta-diversity differences, consistent with the present findings. The absence of significant diversity differences most likely reflects the limited statistical power inherent to the small sample size of the present study, though the stronger confounder control afforded by the twin design may also contribute. All diversity and community structure findings should, therefore, be interpreted as exploratory. In this context. PERMANOVA based on Bray–Curtis dissimilarity identified *Clostridiales* and *Ruminococcaceae* as the taxa contributing most to the observed non-significant variation at the genus level in both cohorts, with *Bacteroides* additionally prominent in the MoCA-discordant group (ACE: *p* = 0.3443; MoCA: *p* = 0.8485).

Regarding taxon-specific findings, the most robust result of the present study is the significantly lower abundance of *Lachnospiraceae* in MCI-affected twins in the ACE-discordant group, identified by ANCOM-BC (log-fold change = −0.041; q = 0.049). This finding is independently corroborated by LEfSe analysis, which also identified reduced *Lachnospiraceae* abundance in ACE-discordant pairs (LDA score = −2.51). The convergence of two methodologically distinct analyses strengthens this observation and is consistent with the broader literature, in which decreased *Lachnospiraceae* abundance has been recurrently associated with cognitive impairment and AD [37,38,39,40]. *Lachnospiraceae* depletion has been linked to reduced short-chain fatty acid availability and downstream disruption of gut epithelial integrity and neuroinflammatory regulation, rendering it a biologically plausible correlate of neurodegeneration [27,39]. Whether this reduction precedes or follows cognitive decline cannot be established from the present cross-sectional data.

ANCOM-BC additionally identified a suggestive trend toward lower *Alphaproteobacteria* abundance in the ACE-discordant group (q = 0.095). Given the absence of consistent prior evidence for this taxon in MCI and the limited sample size of the present study, this finding should be regarded as a preliminary signal requiring independent replication and cannot be meaningfully interpreted in the current state of the literature.

In contrast, LEfSe analysis of the MoCA-discordant group identified higher *Lachnospiraceae* abundance in MCI-affected twins, opposite to the ACE-discordant group. This discrepancy may partly reflect cohort-level heterogeneity arising from the incomplete overlap between subgroups, but it can also highlight a fundamental methodological issue: ACE and MoCA differ in sensitivity profiles, cognitive domains assessed, and classification thresholds. And notably, unlike the MoCA, which incorporates a standardized built-in education-based score correction for participants with 12 or fewer years of formal education, the ACE-III does not apply an equivalent automatic adjustment, although education-stratified cut-off values have been proposed in some validation studies, resulting in partially non-overlapping subpopulations, even within the same cohort. Consequently, the choice of cognitive assessment instrument directly determines which microbiome comparisons are performed. The current microbiome literature lacks a standardized approach to neuropsychological test selection, and the use of different questionnaires may generate divergent results independently of true biological differences. Future studies should explicitly justify instrument selection and, where feasible, apply multiple validated tools in parallel.

LEfSe analysis identified further taxon-level differences of exploratory interest that should be considered exploratory and hypothesis-generating rather than confirmatory findings. In ACE-discordant pairs, reduced *P. copri* abundance was detected alongside higher abundance of *Sutterella*, *Succinivibrio*, *Odoribacter*, *Ruminococcus*, *Ruminococcaceae*, and *Clostridiales* in MCI-affected twins. In MoCA-discordant pairs, *P. copri* showed higher abundance in MCI-affected twins, an opposing direction to that observed in the ACE cohort. This bidirectional pattern is consistent with the complex and context-dependent role of *P. copri* in human diseases [41,42] and is further complicated by the fact that multiple taxonomically distinct *P. copri* entities were identified across cohorts, represented by different reference database identifiers. This likely reflects the known phylogenetic heterogeneity of *P. copri*, whose distinct clades may differ in functional and immunological properties, potentially contributing to the inconsistent findings observed within this study and in the broader literature.

Elevated *Proteobacteria* members, including *Succinivibrio* and *Sutterella*, are broadly consistent with the findings reported by Liu et al. [30] and Hou et al. [43]. An overlap between the two cohorts was observed for *Sutterella*, *Ruminococcus*, and *Clostridiales*, all showing consistently higher abundances in MCI-affected twins across both cognitive assessment groups, albeit at different taxonomic entities. While this convergence may provide some support for these taxa as candidates for future investigation, the limited sample size and exploratory nature of the analysis preclude firm conclusions regarding their role in MCI.

Regarding *Odoribacter*, the existing literature remains small [27], and therefore, the observed association should be interpreted cautiously. The observed elevation is a preliminary signal of uncertain significance and requires independent replication.

The elevation of *Clostridiales* is broadly consistent with prior reports [38], although its functional significance in MCI is not established. The *Ruminococcaceae* results were divergent between cohorts, being higher in ACE-discordant and lower in MoCA-discordant pairs, which is consistent with contradictory data in the literature [37,39], highlighting the uncertainty of these associations and the need for validation in larger cohorts.

Elevated *Erysipelotrichaceae* in MCI-affected twins is of potential interest, given the findings from Zhang et al. [44] in animal models, although extrapolation to human MCI remains speculative.

The detection of elevated *Slackia* is consistent with limited prior evidence of increased *Actinobacteria* in cognitive impairment [37,38], although evidence for this specific genus in MCI remains limited and should be regarded as preliminary. 

*Bacteroides* abundance was elevated in MCI-affected twins in the MoCA cohort, contrasting with the general trend of *Bacteroidetes* reduction reported by Zhuang et al. [37], Li et al. [32], and Pan et al. [34], but consistent with Vogt et al. [45]. Given the exploratory design and small sample size, this discrepancy should not be overinterpreted and may reflect population-level heterogeneity, methodological differences, or stochastic variation.

Across all taxon-level findings, a fundamental interpretive limitation persists: whether the observed gut microbiota alterations contribute to MCI pathogenesis or reflect downstream consequences of cognitive decline cannot be resolved from cross-sectional data. Longitudinal discordant twin designs will be essential to establish temporal precedence.

The primary limitation of this study is the small sample size, which constrains statistical power across all analyses; all findings should, therefore, be regarded as preliminary and exploratory, requiring replication in larger longitudinal twin cohorts. Zygosity was determined by a self-reported questionnaire rather than DNA-based genotyping. The cross-sectional design precludes causal inference, and mixed MCI etiologies may introduce biological heterogeneity despite the exclusion of diagnosed neurological comorbidities.

Several environmental confounders, dietary habits, and physical activity were assessed but not included as covariates, as their inclusion would have further reduced the already limited sample size available for pairwise analysis. Antibiotic, probiotic, and NSAID use were assessed by a self-reported questionnaire and applied as exclusion criteria. However, proton pump inhibitor use, a recognized modifier of gut microbiota composition, was not systematically recorded and represents an uncontrolled source of variability. The self-reported nature of all lifestyle and medication data introduces additional measurement uncertainty.

Finally, the discordance between ACE and MoCA findings in both subgroup composition and taxon-level results underscores that instrument selection is a fundamental methodological variable, directly shaping which individuals are studied and which biological signals are detected, and future studies should standardize and explicitly justify their cognitive assessment approach.

## 5. Conclusions

This is the first gut microbiome study in MCI-discordant monozygotic twin pairs. providing a genetically controlled framework for identifying non-shared biological contributors to cognitive decline. No significant diversity differences were observed, most likely reflecting limited statistical power. The convergent finding of reduced *Lachnospiraceae* abundance in MCI-affected twins across ANCOM-BC and LEfSe is the most robust result, consistent with the broader literature. The remaining findings are exploratory. The divergence between ACE- and MoCA-derived results suggests that cognitive instrument selection may contribute to inconsistencies across the microbiome literature, independent of true biological differences. Whether gut microbiota changes are causally involved in MCI or represent the downstream consequences of cognitive decline remains unresolved, and larger longitudinal twin studies with standardized methodology will be necessary to advance causal inference in this field.

## Figures and Tables

**Table 1:** Descriptive analysis of the two analyzed groups.

	ACE Score Discordant MZ Twins (*n* = 14)	MoCA Score Discordant MZ Twins (*n* = 30)	*p*
Age (years)	71.71 ± 3.27	72.33 ± 2.48	0.48
Gender (males%)	0	20	NA
BMI (kg/m^2^)	26.81 ± 4.19	27.53 ± 4.53	0.61
Smoking (%)	35.71	33.33	1
Hypertension (%)	50	50	1
Cardiovascular disease (%)	7.14	20	0.54
Diabetes (%)	7.14	16.67	0.73
Dyslipidemia (%)	42.86	46.67	1
Sports (%)	42.86	53.33	0.75

Data are presented as mean ± SD for continuous variables and as *n* (%) for categorical variables. The absence of statistically significant differences does not confirm group balance; it reflects only that no significant differences were detected at these sample sizes, which are inherently underpowered for such comparisons. This table is presented for descriptive purposes only and does not constitute a primary analytical comparison. ACE: Addenbrooke’s Cognitive Examination; BMI: body mass index; MoCA: Montreal Cognitive Assessment; MZ: monozygotic; SD: standard deviation.

**Table 2:** Taxa with significantly different relative abundance between MCI-affected and unaffected twins in ACE-discordant monozygotic twin pairs. identified by ANCOM-BC analysis. A positive log-fold change indicates higher abundance in MCI-affected twins; a negative log-fold change indicates lower abundance in MCI-affected twins. Raw *p*-values and adjusted *p*-values (q-values) are presented. Taxa were considered statistically significant at q < 0.05 and suggestive at q < 0.1.

log-Fold Change	*p* Value	q Value	Taxon	Phylum	Class	Order	Family	Genus
−0.0408891448978009	0.000383	0.049	367909	*Firmicutes*	*Clostridia*	*Clostridiales*	*Lachno-* *spiraceae*	NA
−0.00879762620993216	0.001476	0.095	4435655	*Proteobac-teria*	*Alphaproteo-bacteria*	*RF32*	NA	NA

**Table 3:** Taxa identified by LEfSe analysis as differentially abundant between MCI-affected and unaffected twins in ACE- and MoCA-discordant monozygotic twin pairs. Negative LDA scores indicate lower abundance in MCI-affected twins; positive LDA scores indicate higher abundance in MCI-affected twins. The significance threshold for non-parametric tests was set at *p* < 0.1, with a minimum LDA score of 1.0 (log_10_). Taxa are classified by phylum, class, order, family, genus, and species, where available; NA indicates unresolved classification at the given taxonomic level. Taxon identifiers correspond to reference sequences in the 16S rRNA database used for classification. The numbers displayed in the table correspond to taxon reference database identifiers, not quantities. They serve as labels to identify individual taxa in the reference database and do not represent numerical values or counts.

LDA Scores	Taxon	Phylum	Class	Order	Family	Genus	Species
ACE score discordant twins							
−2.50875054982017	367909	*Firmicutes*	*Clostridia*	*Clostridiales*	*Lachnospiraceae*	NA	NA
−1.24589698637538	558839	*Bacteroidetes*	*Bacteroidia*	*Bacteroidales*	*Prevotellaceae*	*Prevotella*	*Copri*
2.04302392675124	189076	*Proteobacteria*	*Beta-proteobacteria*	*Burkholderiales*	*Alcaligenaceae*	*Sutterella*	NA
2.06529539000264	190529	*Firmicutes*	*Clostridia*	*Clostridiales*	NA	NA	NA
2.09089604390726	228199	*Firmicutes*	*Clostridia*	*Clostridiales*	*Rumino-coccaceae*	*Ruminococcus*	NA
2.1931422676267	163857	*Proteobacteria*	*Gamma-proteobacteria*	*Aeromonadales*	*Succini-vibrionaceae*	*Succinivibrio*	NA
2.27666410554975	4454586	*Bacteroidetes*	*Bacteroidia*	*Bacteroidales*	*Odori-bacteraceae*	*Odoribacter*	NA
2.36177810006473	344804	*Firmicutes*	*Clostridia*	*Clostridiales*	*Rumino-coccaceae*	NA	NA
MoCA score discordant twins							
−1.72748190567828	317135	*Firmicutes*	*Clostridia*	*Clostridiales*	*Rumino-coccaceae*	NA	NA
1.02107801607861	180107	*Firmicutes*	*Clostridia*	*Clostridiales*	*Rumino-coccaceae*	*Ruminococcus*	NA
1.29220178370222	361727	*Firmicutes*	*Clostridia*	*Clostridiales*	NA	NA	NA
1.30484078678649	545061	*Bacteroidetes*	*Bacteroidia*	*Bacteroidales*	*Prevotellaceae*	*Prevotella*	*Copri*
1.35967730361813	287508	*Actinobacteria*	*Coriobacteria*	*Coriobacteriales*	*Corio-bacteriaceae*	*Slackia*	NA
1.49772968055921	369027	*Firmicutes*	*Clostridia*	*Clostridiales*	*Lachnospiraceae*	NA	NA
1.54965399803863	174978	*Bacteroidetes*	*Bacteroidia*	*Bacteroidales*	*Bacteroidaceae*	*Bacteroides*	NA
1.64609350991407	194360	*Proteobacteria*	*Beta-proteobacteria*	*Burkholderiales*	*Alcaligenaceae*	*Sutterella*	*NA*
1.64957614245984	509416	*Firmicutes*	*Clostridia*	*Clostridiales*	NA	NA	NA
1.69016184449878	580008	*Firmicutes*	*Erysipelotrichi*	*Erysipelo-trichales*	*Erysipelo-trichaceae*	NA	NA
1.84466841210673	357312	*Firmicutes*	*Clostridia*	*Clostridiales*	*Lachnospiraceae*	NA	NA
2.13502267125564	292921	*Bacteroidetes*	*Bacteroidia*	*Bacteroidales*	*Prevotellaceae*	*Prevotella*	*Copri*

## Data Availability

The datasets analyzed during the current study are not publicly available because they are part of ongoing and planned research projects. However, anonymized data are available from the corresponding author upon reasonable request, subject to ethical approval and applicable data protection regulations.

## Supplementary Materials

The following supporting information can be downloaded at https://www.mdpi.com/article/10.3390/medicina62061106/s1: Figure S1: Beta diversity assessed by weighted UniFrac dissimilarity-based Principal Coordinates Analysis (PCoA) in MCI-discordant monozygotic twin pairs at genus level. Panel A: ACE-discordant pairs (*n* = 7); Panel B: MoCA-discordant pairs (*n* = 15). No distinct clustering by MCI status was observed in either cohort.

## Abbreviations

ACE	Addenbrooke’s Cognitive Examination
AD	Alzheimer’s disease
ANCOM-BC	Analysis of Composition of Microbiomes with Bias Correction
LDA	Linear Discriminant Analysis
LEfSE	Linear Discriminant Analysis Effect Size
MCI	Mild cognitive impairment
MoCA	Montreal Cognitive Assessment
MZ	Monozygotic
NGS	Next-generation sequencing
OTU	Operational taxonomic unit
PCoA	Principal coordinate analysis
PERMANOVA	Permutational Multivariate Analysis of Variance
