# Supplementary figures and images for: The Role of Gut Microbiome in Mild Cognitive Impairment: A Twin Study
Source: Medicina (Kaunas). 2026 Jun 6;62(6):1106. doi: 10.3390/medicina62061106 (PMC13302825; doi:10.3390/medicina62061106)

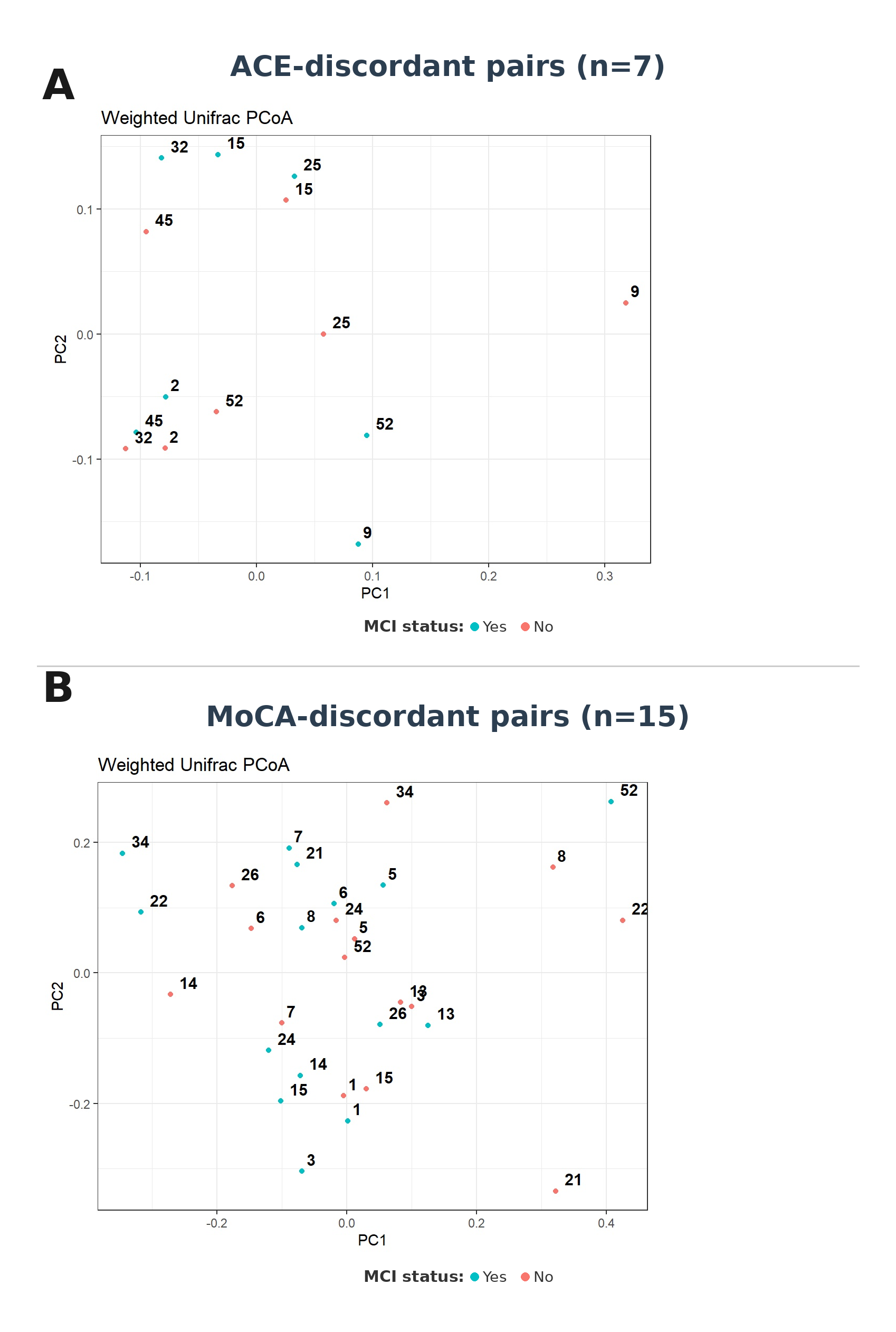

Supplement: Supplementary file 1 [file medicina-62-01106-s001.zip › medicina-4130286-supplementary.tiff]
